# Case Report: A novel strategy for superior petrosal vein management in cerebellopontine angle surgery

**DOI:** 10.3389/fonc.2026.1754539

**Published:** 2026-05-14

**Authors:** Ligang Chen, Xinyu Yang, Xuantong Liu, Yinuo Wang, Geyu Wang, Xinning Li, Xiaoyu Sun, Sizhe Feng

**Affiliations:** 1 Department of Neurosurgery, General Hospital of Northern Theater Command, Shenyang, China; 2 Department of Pathology, General Hospital of Northern Theater Command, Shenyang, China; 3 Department of Plastic Surgery, General Hospital of Northern Theater Command, Shenyang, China

**Keywords:** case report, superior petrosal vein, surgical technique innovation, venous collateral pathways, venous flow redistribution

## Abstract

The superior petrosal vein (SPV) is a vital venous drainage system in the posterior fossa. It is often sacrificed during cerebellopontine angle (CPA) surgery to enhance surgical exposure, leading to postoperative complications. This study introduces the “Venous Flow Redistribution” (VFR) technique, a novel approach that preserves the SPV’s main venous trunk while severing its distal connections, thus enabling optimal tumor exposure and minimizing venous injury. We present a case of right-sided CPA meningioma treated with suboccipital retrosigmoid craniotomy and the VFR technique. Postoperatively, the patient had an uneventful recovery with improvement in headache and hearing loss, without any complications. The VFR technique leverages multiple venous collateral pathways, redistributing venous flow and maintaining drainage, thereby enhancing surgical flexibility while reducing the risk of complications. This method might be a promising strategy for SPV preservation during complex CPA surgeries.

## Introduction

The petrosal veins are essential venous drainage systems in the posterior fossa, classified as either superior petrosal veins (SPVs) or inferior petrosal veins based on their entry points into the superior or inferior petrosal sinuses, respectively (1). The SPVs originate from the cerebellopontine angle cistern, receiving tributaries from the pontine transverse veins, superior vermian veins, hemispheric superior cerebellar veins, horizontal fissure veins, and veins of the fourth ventricular recess (2, 3). Most of these branches converge into a single main trunk before entering the superior petrosal sinus; however, in some cases, they form two or three trunks that enter the sinus at different points. These veins have short, thin-walled trunks that serve as crucial drainage pathways for the brainstem and cerebellum (4). Due to their proximity to critical neurovascular structures, surgeons often have to manage these veins during tumor resection (5). Traditional surgical approaches typically require the sacrifice of the SPVs to enhance the surgical view, which can lead to complications such as cerebellar edema, hemorrhagic infarction, and brainstem compression (6, 7). Therefore, preserving the SPVs is paramount during cerebellopontine angle (CPA) tumor surgery (8). Koerbel et al. (9) have demonstrated that postoperative venous-related complications could be as high as 30 when a petrosal vein was occluded. Thus, developing surgical techniques that protect the SPVs has become a key focus during CPA tumor surgery. Recent advancements in surgical methods have allowed for better SPV preservation. Building on our previous experience, we developed a novel surgical strategy known as “Venous Flow Redistribution” (VFR). This innovative technique utilizes multiple venous drainage pathways within the intracranial venous system, preserving the SPV trunk while severing distal connections to achieve full tumor exposure with minimal venous injury.

## Case description

A 61-year-old female presented with persistent headache and progressive hearing loss in her right ear. The patient had not received any previous treatment. Physical examination revealed severe decline in hearing on the right side. The patient had no history of major surgeries or family history of neurological disorders.

**Figure 1:**
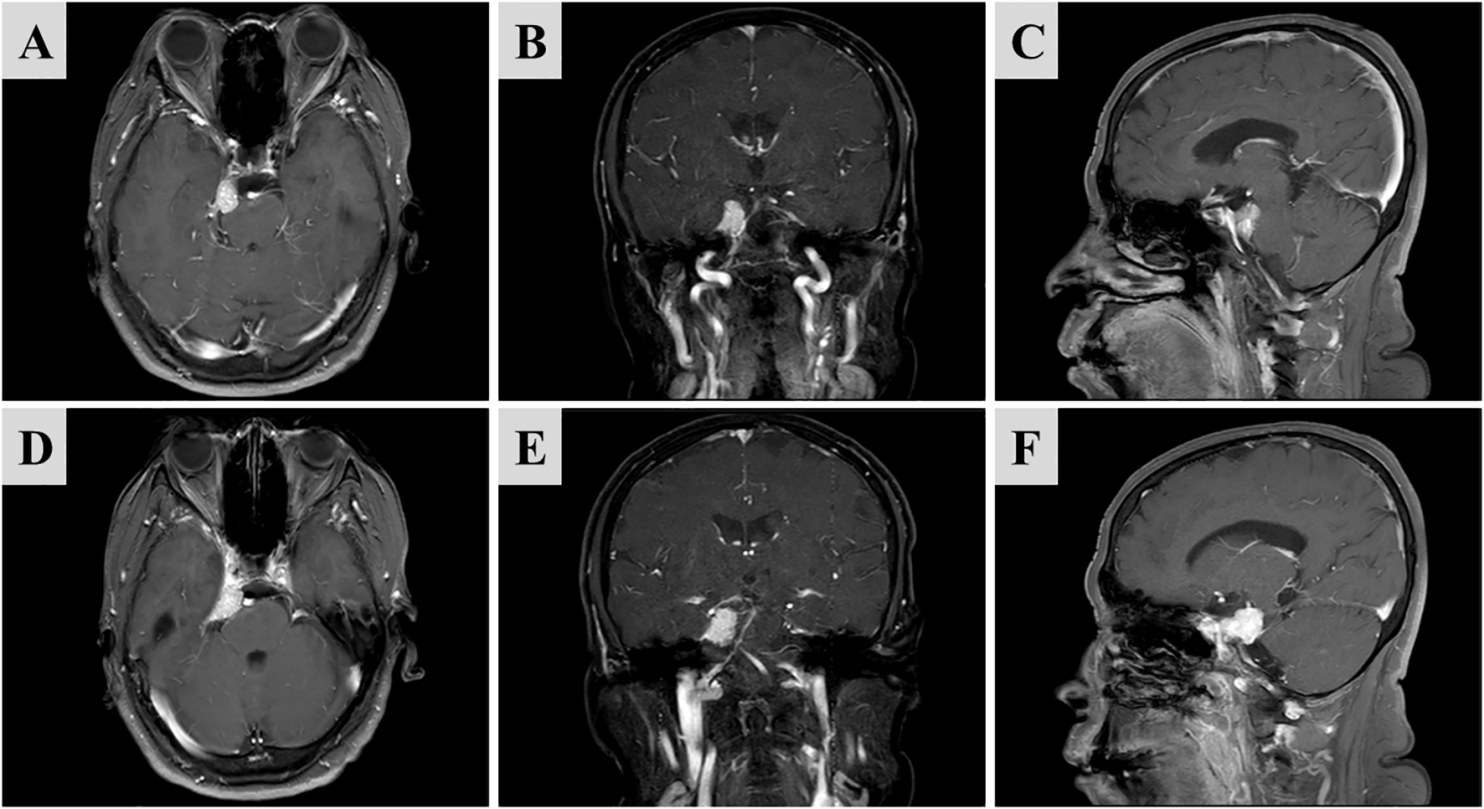
Preoperative contrast-enhanced MRI demonstrating a mass in the right cerebellopontine angle. Homogeneous enhancement is visible at the right petrous apex. The dural tail sign could be seen on the axial plane. **(A, D)** Axial; **(B, E)** Coronal; **(C, F)** Sagittal.

Magnetic resonance imaging (MRI) with contrast enhancement revealed a well-defined tumor in the right cerebellopontine angle, measuring approximately 3.2 cm in diameter. The lesion showed uniform and intense enhancement, leading to an initial diagnosis of a petrous apex meningioma (**Figure 1**), which helps differentiate it from vestibular schwannoma.

**Figure 2:**
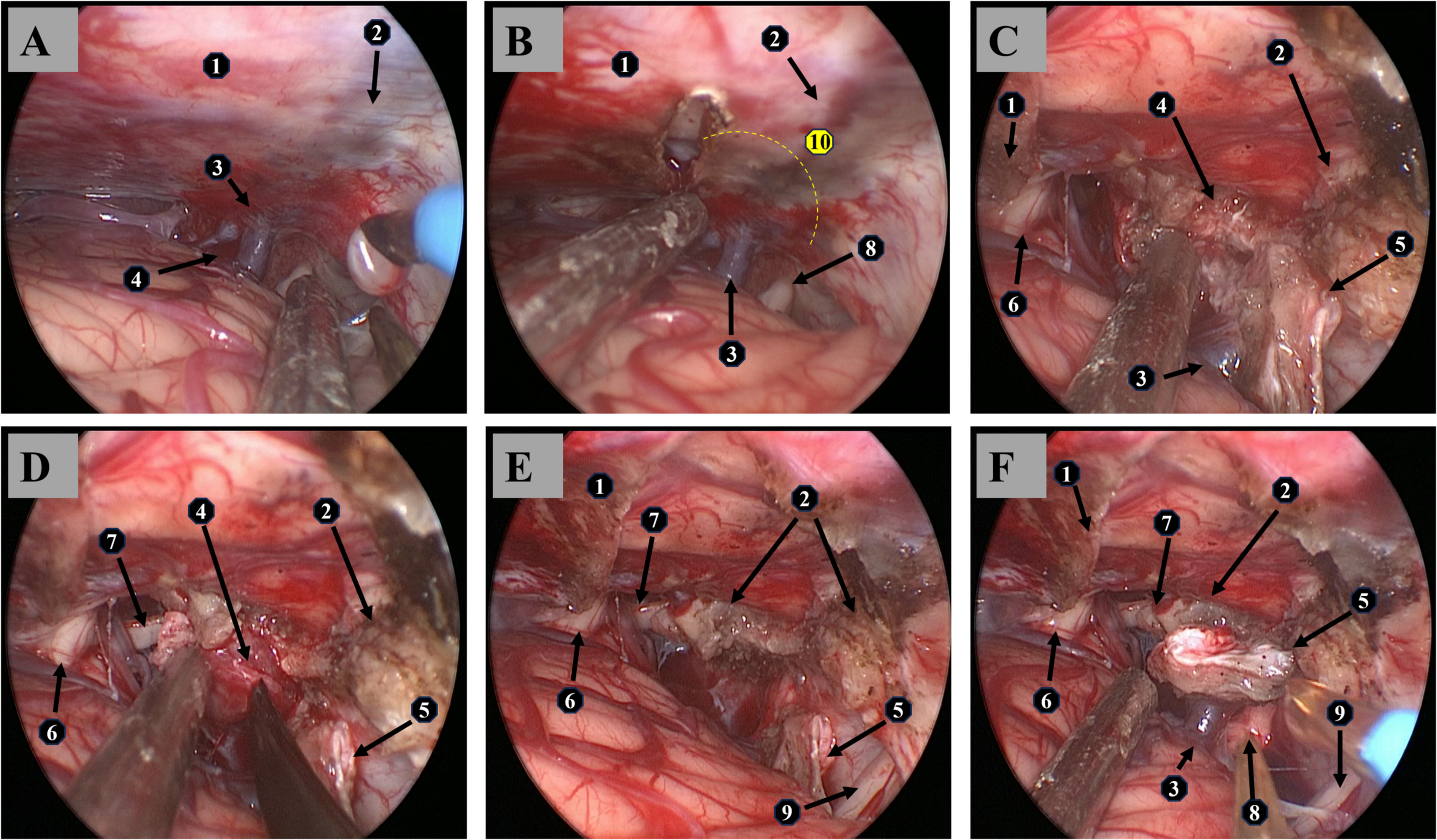
Surgical procedure. **(A)** Endoscopic view through a right retrosigmoid approach, utilizing appropriate patient positioning and intraoperative cerebrospinal fluid release to expose the tumor and superior petrosal vein. **(B)** Under endoscopic guidance, the tentorium was incised, expanding the surgical field and creating working space, while partially interrupting the tumor’s blood supply through the tentorium. **(C)** Multiple tributaries of the superior petrosal vein converge into a single trunk that drains into the superior petrosal sinus. The main SPV trunk was severed intraoperatively, preserving the confluence. The distal end of the severed SPV was carefully coagulated, and the vein was gently retracted to expose the tumor. **(D)** Tumor resection was performed with meticulous protection of adjacent neurovascular structures. **(E)** Complete tumor resection was achieved, and hemostasis was secured with increased pressure control. **(F)** Post-tumor resection, the right petrous apex is visible, along with the free-floating superior petrosal vein. ①Tentorium cerebelli. ②Right superior petrosal sinus. ③Superior petrosal vein. ④Tumor. ⑤Free-floating distal end of the superior petrosal vein trunk. ⑥Brainstem. ⑦Oculomotor nerve. ⑧Trigeminal nerve. ⑨Facial nerve. ⑩Superior petrosal vein transection site (indicated by the dashed line).

The patient underwent a lateral suboccipital retrosigmoid craniotomy. During the surgery, the superior petrosal vein (SPV) trunk was visualized and dissected under endoscopy. The “Venous Flow Redistribution” (VFR) technique was employed. Using meticulous microsurgical techniques, the SPV trunk was exposed and preserved, ensuring no direct injury to the vein. A large SPV was identified anterior to the tumor, where multiple venous branches converged into a single trunk and then drained into the superior petrosal sinus, which obstructed the surgical approach (**Figure 2A**). The nearby tentorium was carefully incised, and the distal SPV (at its connection to the superior petrosal sinus) was severed, while the proximal confluence was preserved, ensuring an unobstructed surgical view (**Figures 2B, C**). With optimal exposure, the tumor was completely resected (**Figures 2D, E**). The preserved SPV confluence and severed distal end were clearly visible (**Figure 2F**). Intraoperative electrophysiological monitoring was employed to ensure maximal tumor resection while preserving cranial nerve integrity.

**Figure 3:**
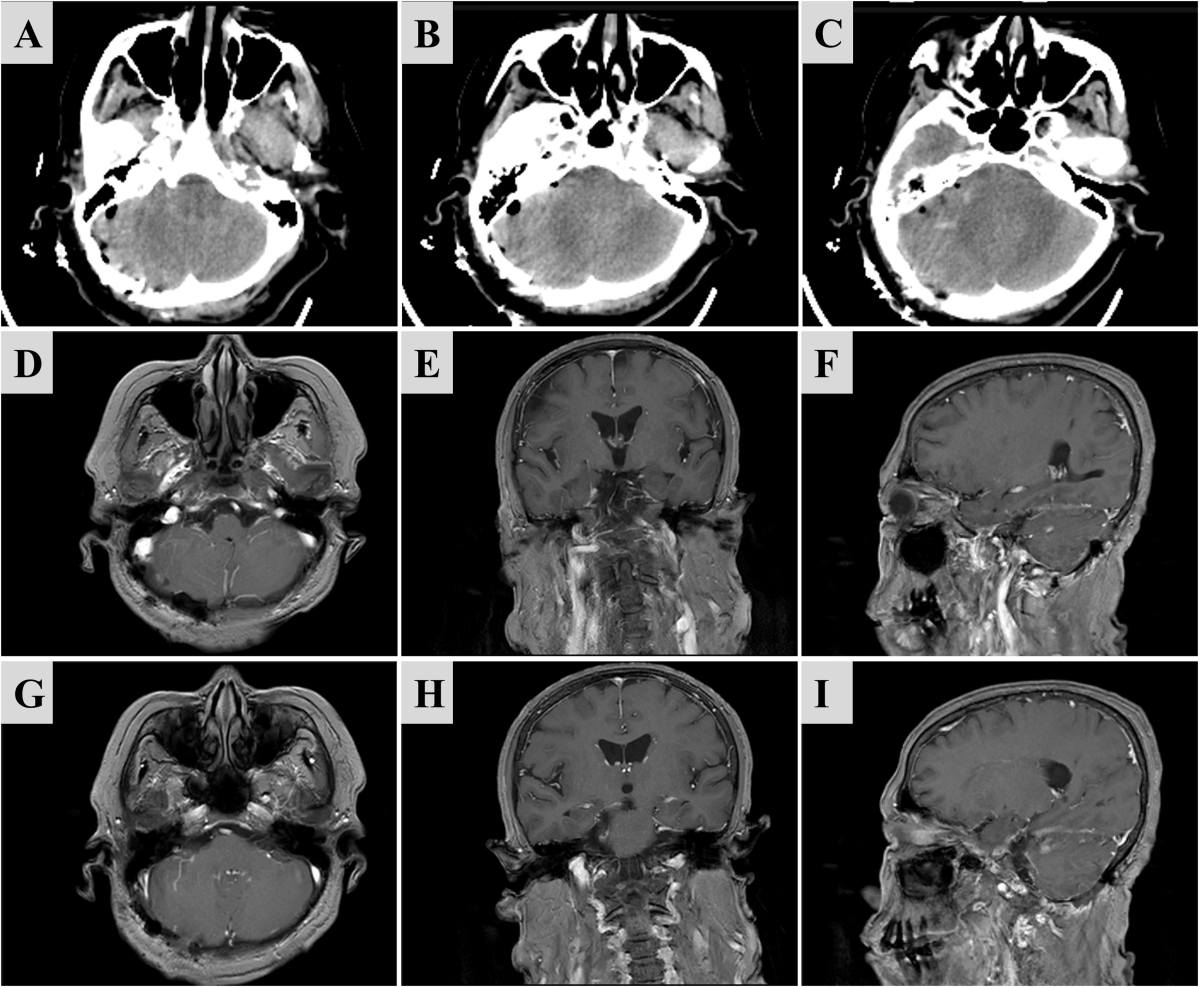
Immediate postoperative CT demonstrated gross total resection of the tumor **(A–C)**. Postoperative contrast-enhanced MRI at follow-up showing gross total resection of the tumor **(D–I)**.

**Figure 4:**
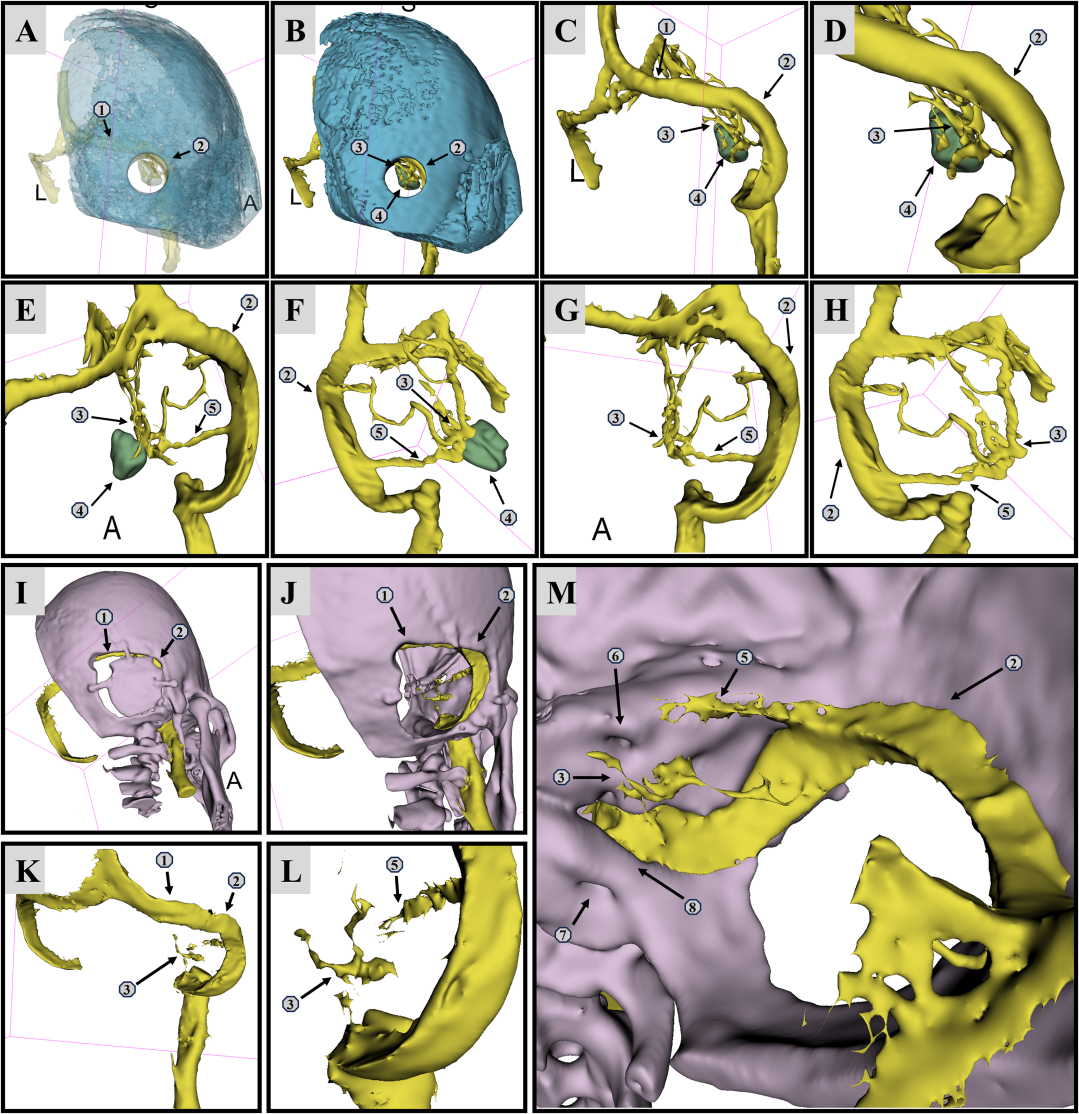
Preoperative CTA reconstruction showing the tumor and superior petrosal vein. **(A–D)** The superior petrosal vein is located posterior to the tumor, obstructing the surgical view in the simulated retrosigmoid approach. **(E–H) **Multiple tributaries of the superior petrosal vein converge into a single trunk, draining into the superior petrosal sinus. **(I, J)** The bone window position for the right retrosigmoid approach. **(K–M)** The superior petrosal vein treated using the VFR technique is clearly visualized, with its free-floating distal end now distant from the superior petrosal sinus, positioned at the level of the internal auditory canal. This confirms that the preserved venous tributaries are still functioning in drainage. ①Right transverse sinus. ②Right sigmoid sinus. ③Petrosal vein. ④Tumor location. ⑤Right superior petrosal sinus. ⑥Internal auditory canal. ⑦Hypoglossal canal. ⑧Jugular foramen.

Histopathological examination confirmed the final diagnosis of meningioma (WHO Grade I). Postoperatively, the patient reported improved hearing and complete resolution of headache symptoms. Immediate postoperative CT demonstrated gross total resection of the tumor (**Figures 3A–C**). At the 3-month follow-up, MRI showed gross total resection of the tumor (**Figures 3D–I**). Preoperative and postoperative CTA reconstructions demonstrated significant anatomical repositioning of the superior petrosal vein. The free-floating SPV trunk and its branches had moved away from the superior petrosal sinus, now draping along the cerebellar surface at the level of the internal auditory canal (**Figure 4**). No postoperative complications were reported.

## Discussion

The superior petrosal veins (SPVs), also known as Dandy’s veins (10), form a crucial venous system within the cerebellopontine angle (CPA). They originate from the transverse cerebellopontine fissure and receive multiple tributaries from the brainstem, including the transverse pontine veins, superior vermian veins, and veins of the fourth ventricular recess. These veins ultimately drain into the superior petrosal sinus (SPS) (2, 11). The primary function of the SPVs is to drain blood from the cerebellum, brainstem, and temporal lobe. Typically, the SPVs converge into a single trunk, though in some cases, two or three trunks may form and drain separately into the SPS (1).

Due to their proximity to critical neurovascular structures, the complex anatomy of the SPVs requires careful preservation during surgery. In neurosurgical procedures, particularly during approaches such as the subtemporal transtentorial or retrosigmoid suprameatal routes, anatomical variations in SPV drainage play a critical role in determining surgical strategy and safety. The SPV drainage pattern can vary significantly between individuals. Based on the relationship between the SPVs’ entry into the SPS, the Meckel’s cave, and the internal auditory canal (IAC), the SPV drainage pattern can be classified into three types (12):

- Type I: The SPV drains into the SPS above and lateral to the IAC.
- Type II: The most common type, where the SPV drains between the lateral border of the trigeminal nerve entering Meckel’s cave and the medial border of the facial nerve entering the IAC, typically within a 13 mm range.
- Type III: The SPV drains above or medial to Meckel’s cave, situated on the medial side of the lateral border of the trigeminal nerve entering Meckel’s cave.

In a subtemporal transtentorial approach, more surgical space could be gained with the Type I SPV, however, surgical exposure may be significantly limited in case of Type III and specific Type II patterns (12). During tumor resection, the SPVs often obstruct the surgical view, and sometimes the sacrifice of them is a precondition for tumor resection (13). However, this maneuver can lead to several complications, including venous infarction, hemorrhagic edema, neurological damage, and even life-threatening brainstem compression (7).

Matsushima et al. (14) proposed a classification system for the SPVs based on their drainage pathways: anterior pontomesencephalic, posterior pontomesencephalic, tentorial, and petrosal groups. The transverse cerebellopontine fissure vein is the largest tributary of the SPVs, and understanding its anatomy is critical in avoiding major venous complications during petrous apex meningioma surgery (15). During surgery, it is recommended to first identify the transverse cerebellopontine fissure vein and expose it along its course to ensure venous protection throughout the procedure. This strategy primarily focuses on protecting the SPVs, but in some surgical approaches, such as the subtemporal transtentorial route or the retrosigmoid suprameatal route, the size and position of the SPVs may affect the extent of the surgical field. If the vein is severed, additional surgical maneuvers may be required to control bleeding, potentially increasing operative time and complexity. To address this challenge, we propose a new solution based on the dynamic multi-pathway characteristics of intracranial venous hemodynamics (1): the VFR technique. The intracranial venous system is not solely reliant on a single, fixed drainage route; instead, it has multiple collateral and interconnecting drainage pathways. Taking advantage of this feature, the proximal trunk of the SPVs after confluence can be preserved, while the distal portion connecting to the SPS is severed. This keeps the SPV trunk free during surgery, allowing better exposure of the tumor (**Figure 2**). After severing the connection between the trunk and the superior petrosal sinus, blood flow is redistributed through collateral channels from several SPV tributaries, ensuring effective drainage of the relevant regions. This flow redistribution process maintains hemodynamic balance and provides greater flexibility during surgery. By optimizing and redistributing venous drainage pathways, this technique adapts to complex surgical procedures without sacrificing major venous function, thereby avoiding the related complications.

The superior petrosal veins (SPVs) drain into the superior petrosal sinus after converging from several tributaries, resembling the way an octopus’s tentacles converge toward its center. The Venous Flow Redistribution (VFR) technique works by severing the distal connections of the SPVs while preserving the main venous trunk, allowing blood flow to be redistributed through collateral channels. This approach has been shown to reduce the incidence of postoperative complications. Additionally, by dynamically adjusting the venous drainage pathways, the VFR technique not only maximizes the surgical field but also ensures proper venous function postoperatively.

There are several limitations in this case report. First, postoperative CTA reconstruction only demonstrated the existence of blood flow in the SPV, however, cerebral angiography is further necessitated to verify the redistributed venous flow in the SPV. Second, more clinical cases are needed to validate its efficacy in CPA tumors resection. Third, a longer-term follow-up should be performed to monitor the blood flow status of the SPV and postoperative complications of this technique.

## Conclusion

In certain specialized surgical procedures where the superior petrosal vein cannot be preserved, it becomes necessary to identify an appropriate site for division. The VFR technique offers an entirely novel approach to dividing the superior petrosal vein: it preserves the normal distribution between branches, allowing venous blood to redistribute between them. The VFR technique provides a safer and more flexible surgical field while maintaining venous drainage function, possibly by leveraging the natural collateral pathways of the intracranial venous system, thus reducing the risk of postoperative complications. We believe this technique might be an ultimate piece of the surgeon’s armamentarium to improve outcomes in CPA tumors.

The VFR technique represents a significant innovation in venous preservation during complex skull base surgeries, particularly those involving the superior petrosal veins (SPVs). By leveraging the natural collateral pathways of the intracranial venous system, the VFR technique allows for selective severance of distal venous connections while preserving the proximal venous trunk. This not only maintains venous drainage function but also provides a safer and more flexible surgical field, ultimately reducing the risk of postoperative complications, such as venous infarction, edema, and brainstem compression.

The ability to dynamically redistribute venous flow using the VFR technique offers surgeons greater control over challenging tumor resections in the cerebellopontine angle (CPA) region. It mitigates the traditional need to sacrifice critical veins that would otherwise obstruct the operative view, providing a balance between achieving optimal tumor exposure and preserving vital venous structures. This adaptability, combined with the minimization of venous injury, positions the VFR technique as a promising advancement in neurosurgical procedures.

As more clinical cases and research studies continue to validate its efficacy, the VFR technique holds the potential to become a standard approach not only for CPA tumors but also for a wide range of neurosurgical interventions requiring precise venous management. Future studies are anticipated to further explore its applicability in surgeries involving venous drainage preservation and to refine the technique for broader use in both skull base and cranial surgeries. The evolution of venous preservation strategies, such as VFR, will likely pave the way for safer, more effective treatments for patients undergoing complex intracranial operations.

This study has several limitations. First, the evidence for venous flow redistribution is indirect, derived from postoperative CTA demonstrating SPV trunk patency and uneventful clinical recovery, rather than direct hemodynamic assessment. We acknowledge the lack of intraoperative ICG angiography or pre- and postoperative DSA/MRV to objectively confirm collateral recruitment and quantify flow dynamics. Consequently, we cannot exclude the possibility of preoperative compensatory shunting due to chronic tumor compression. Second, as a single-case report, the generalizability of this technique remains to be established. Future studies will incorporate intraoperative ICG fluorescence angiography to visualize real-time tributary filling, as well as pre- and postoperative MRV or DSA to validate collateral pathway recruitment in a larger cohort.

## Data Availability

The datasets presented in this article are not readily available because of ethical and privacy restrictions. Requests to access the datasets should be directed to the corresponding author.
